# Skeletal Muscle-derived Hematopoietic Stem Cells: Muscular Dystrophy Therapy by Bone Marrow Transplantation

**DOI:** 10.4172/2157-7633.S11-005

**Published:** 2012-11

**Authors:** Atsushi Asakura

**Affiliations:** Stem Cell Institute, Paul and Sheila Wellstone Muscular Dystrophy Center, Department of Neurology, University of Minnesota Medical School, Minneapolis, MN, USA

**Keywords:** Muscle stem cells, Hematopoietic stem cell, Hematopoiesis, Satellite cell, Side population, Skeletal muscle, Muscular dystrophy

## Abstract

For postnatal growth and regeneration of skeletal muscle, satellite cells, a self-renewing pool of muscle stem cells, give rise to daughter myogenic precursor cells that contribute to the formation of new muscle fibers. In addition to this key myogenic cell class, adult skeletal muscle also contains hematopoietic stem cell and progenitor cell populations which can be purified as a side population (SP) fraction or as a hematopoietic marker CD45-positive cell population. These muscle-derived hematopoietic stem/progenitor cell populations are surprisingly capable of differentiation into hematopoietic cells both after transplantation into irradiated mice and during *in vitro* colony formation assay. Therefore, these muscle-derived hematopoietic stem/progenitor cells appear to have characteristics similar to classical hematopoietic stem/progenitor cells found in bone marrow. This review outlines recent findings regarding hematopoietic stem/progenitor cell populations residing in adult skeletal muscle and discusses their myogenic potential along with their role in the stem cell niche and related cell therapies for approaching treatment of Duchenne muscular dystrophy.

## Muscle Satellite Cells

Myogenic satellite cells are a stem cell population that contributes to postnatal muscle growth and regeneration that reside beneath the basal lamina of adult skeletal muscle, closely juxtaposed to the muscle fibers. Satellite cells are normally mitotically quiescent, but following injury or exercise, they initiate proliferation and give rise to daughter myogenic precursor cells [1–3]. After multiple rounds of cell division, these myogenic precursor cells exit their cell cycle and fuse with each other to terminally differentiate into multinucleated myotubes. The self-renewal capacity within the satellite cell compartment was proven when considering the fact that the number of quiescent satellite cells in adult muscle remains relatively constant over multiple cycles of degeneration and regeneration [4,5]. In addition, recent work demonstrates that a small number of satellite cells can robustly contribute to regenerating muscle maintenance of the satellite cell compartment, confirming the proof of concept for stemness of satellite cells [6].

A decade ago, satellite cells were considered monopotent stem cells, with the ability to give rise only to cells of the myogenic cell lineage. Indeed, both quiescent satellite and myogenic precursor cells express markers for myogenic cells such as M-cadherin, Pax3, Pax7, and Myf5 during the quiescent state, and M-cadherin, Pax7, Myf5, MyoD and desmin during myogenic proliferation [7–11]. However, recent experiments have demonstrated that satellite cells possess multipotential differentiation capability. Upon induction, satellite cells are capable of differentiation into adipocytes and osteocytes *in vitro* [9,12] and fibroblast *in vivo* [13], indicating a mesenchymal differentiation potential of satellite cells. However, *in vivo* situation, the ability of adipogenic or osteogenic potential for satellite cells is very limited, and satellite cells may only contribute to skeletal myogenesis in normal situation [14–16]. More recently, satellite cells have been induced to generate induced Pluripotent Stem (iPS) cells by transduction of iPS cell-inducing transcription factors, Oct4, Sox2, cMyc and Klf4 [17–20].

## Muscle-derived HSCs

Current work demonstrates that adult skeletal muscle-derived cells exhibit the capacity to reconstitute the entire hematopoietic repertoire following intravenous injection into lethally irradiated mice [21–29]. Myogenic cells have also been found to form multiple types of hematopoietic colonies by *in vitro* hematopoietic colony forming assay [25–30]. However, these muscle-derived hematopoietic stem cells (HSCs) and hematopoietic progenitor cells (HPCs) were confirmed as a distinct population from satellite cells [30].

During mouse embryogenesis, the process of primitive hematopoiesis begins in the yolk sac on embryonic day 7.5 (E7.5). Thereafter, definitive HSC activity is first detectable in the aortagonad-mesonephros (AGM) region on E10, and then fetal liver and yolk sac. Subsequently, the fetal liver becomes the main tissue for definitive hematopoiesis by E12. During late embryogenesis, the HSC population in the fetal liver migrates to the bone marrow, which then remains the major site of hematopoiesis throughout adult life [31]. In adult, HSCs and HPCs originating from bone marrow readily colonize the adult spleen. However, it was initially controversial whether HSCs/HPCs exist outside of bone marrow or spleen. Bartlett [32,33] first reported that a significant amount of hematopoietic colony forming units(spleen, CFU-s), were present in mouse adult brain. The average number of CFU-s obtained per dissociated adult brain-derived cells was significantly higher than those of other adult tissues including lung, kidney, heart, thymus and blood. However, Hoogerbrugge et al. [33] failed to obtain such high number of CFU-s in adult brain. Therefore, they concluded that the CFU-s detected by Bartlett in preparations of mouse brain did not originate from the brain tissue. Recent work has challenged this question and revealed that HSCs/HPCs clearly exist in several adult tissues besides bone marrow and spleen [30,34]. For example, not only fetal liver but also adult liver has been shown to contain HSCs that reconstitute the entire hematopoiesis lineage in lethally irradiated animals [35,36]. In addition, adult lung contains large numbers of alveolar macrophages derived from progenitors [37]. Furthermore, T cell differentiation occurs in extra-thymic sites, such as intestine and liver [38,39]. For teleosts (fishes), the kidneys are the major hematopoietic organs containing hematopoietic stem cells which are able to be fractionated as side population (SP) cells, and can give rise to all lines of hematopoietic differentiation including erythropoiesis, granulopoiesis, and lymphopoiesis [40,41]. Finally, it was also reported that adult skeletal muscle too contains HSCs and HPCs [21,23].

HSCs in adult skeletal muscle were first discovered by Gussoni et al. [21]. Gussoni et al. [21] purified SP cells positive for HSC marker Sca-1 from adult skeletal muscle, intravenously injected these muscle SP cells into lethally irradiated mice, and observed whole hematopoietic contribution in the recipient mice. These resultant data strongly indicated that muscle SP fraction contains HSCs. SP cells exclude Hoechst 33342 DNA-binding dye through the activity at the cell surface of multi-drug resistance (MDR) pomp proteins such as ABCG2/BCRP1(see in review) [1], which was first reported by Goodell et al. [42]. They also discovered that HSCs in bone marrow from many different species can be isolated as SP cells by fluorescence activated cell sorting (FACS). *In vitro* hematopoietic colony formation assays confirmed that adult muscle contains a remarkably high level of HPCs that differentiate into multiple types of hematopoietic colonies including myeloid cells, B cells and erythrocytes (see in review) [1,26,30,34,40,43,44]. These muscle-derived HPCs can also be enriched in the muscle SP fraction as they are in bone marrow-derived SP cells [30,34,40,43]. In addition, only CD45(+) muscle-derived cells display the capacity to give rise to hematopoietic cells *in vitro* and reconstitute the entire hematopoietic repertoire following intravenous injection into lethally irradiated mice [1,21,24,45], strongly indicating that muscle-derived HSCs and HPCs are indeed of bone-marrow origin. Therefore, circulating HSCs and HPCs originating from bone marrow may reside within skeletal muscle during developmental stages. In this case, marrow-derived cells migrate into skeletal muscle via activity of hepatocyte growth factor (HGF) and its receptor, c-met [46]. Interestingly, Single-cell-sorted muscle SP/CD45(+) cells displayed robust proliferative activity [29]. These amplified clonal cell populations displayed multilineage differentiation capability, including myeloid, lymphoid and NK cells. Therefore, similar to bone marrow-derived cells, a single cell in muscle-derived hematopoietic cells exhibits major proliferative potential and multi-lineage differentiation capability.

With the current understanding of muscle-related hematopoietic status, there are several intriguing questions. 1) How does the muscle niche maintain HSCs/HPCs that possess such a remarkable capability for hematopoietic differentiation potential? 2) Can muscle-derived HSCs/HPCs contribute to muscle regeneration through their direct myogenic differentiation? 3) Can bone marrow-derived HSCs/HPCs contribute to regenerating muscle fibers through their direct myogenic differentiation?

For the muscle niche, a recent paper showed that HPCs occurred in cachectic muscle with a statistically significant enrichment in Sca-1(+) CD45(+) [47]. Since HSC recruitment is stimulated by muscle injury or other insults [48–50], this phenomenon can be interpreted as a response to signals released by the atrophying fibers to maintain HPCs. Muscle-derived HSCs/HPCs must be abundant in muscle on a whole body basis since muscle is the largest tissue set in the body. Interestingly, Tsuboi et al. demonstrates that frequency of hematopoietic stem cells in human muscle is approximately four times greater than in peripheral blood, suggesting an additional function of human skeletal muscle as a reservoir of HSCs [51]. The presence of HSCs/HPCs in adult muscle raises the possibility that such stem cells locally contribute to host myogenesis when exposed to the correct environment during regeneration. In addition, an interesting question is to what extent non-satellite cells, including muscle-derived HSCs and HPCs, can contribute to regenerating muscle fibers in normal and diseased muscle. Recent work clearly demonstrates that the muscle-derived HSCs/HPCs have been shown to possess myogenic potential, and to contribute to muscle repair by low-level fusion into multinucleated muscle fibers. Regenerative signals in the muscle recruit resident muscle-derived HSCs or HPCs to progress down a myogenic lineage through Wnt signaling and subsequent Pax7 expression [49,52], indicating the participation of muscle-derived HSCs/HPCs in myogenic regeneration.

## Myogenic Contribution of HSCs

Currently, muscle-derived HSCs/HPCs are believed to originate from bone marrow and probably from homing cells of circulating HSCs/HPCs. Several papers have demonstrated the myogenic contribution of bone marrow-derived HSCs/HPCs after intramuscular or intravenous transplantation [21,50,53–64]. Strikingly, single HSC transplantation into lethally irradiated mice demonstrates the clear myogenic contribution of HSCs through intermediate stage myeloid cell differentiation of the engrafted HSCs [56,60–68]. In these cases, the ongoing muscle regeneration and inflammatory cell infiltration are required for HSC-derived contribution. Interestingly, bone marrow-derived CD45(+)/Sca-1(+) cells carrying reporter genes controlled by muscle-specific regulatory elements from the Myf5, myosin light chain (MLC3F), or MCK genes, are induced by myoblasts to activate muscle-specific genes [69]. However, these cells undergo incomplete myogenic specification and differentiation independently from Pax7 and MyoD. Analysis of muscle chimerism in unirradiated animals joined surgically by parabiosis revealed that contributions of circulating cells to myofibers in the skeletal muscle are injury-dependent and that at least some circulating cells have the potential to contribute to regenerating muscle derived from bone marrow [48]. There are two potential mechanisms for the adoption of a myogenic differentiation fate by the progeny of HSCs. First, myogenic differentiation potential of HSCs could be induced by local muscle environments. Alternatively, the formation of heterokaryons between HSCs and myoblasts and/or regenerating muscle fibers through cell fusion could lead to nuclear reprogramming [60]. Latter case is most likely since circulating HSC-derived myeloid progenitor cells, in response to inflammatory cues, migrate to regenerating skeletal muscle and stochastically incorporate into mature myofibers possibly by direct cell fusion process. Potential mechanism for fusion process of myeloid cells may be mediated by the fusogenic ability of macrophages [56]. However, more primitive HSC derivatives, such as myelomonocytic progenitors, but not CD11b-Cre-positive macrophages, neutrophils and natural killer cells, can incorporate into regenerating muscle fibers [68]. Therefore, in the future, the exact mechanism for this heterokaryon formation should be elucidated.

The Kuwana et al. [70] reported the discovery of a primitive cell population termed monocyte-derived multipotential cells (MOMCs), which has a fibroblast-like morphology in culture and a unique phenotype positive for CD14, CD45, CD34 and type I collagen, and were found to originate from HSC-derived circulating CD14(+) monocytes. MOMCs contain progenitors with capacity to differentiate into a variety of nonphagocytes, including bone, cartilage, fat, skeletal and cardiac muscle, neuron, and endothelium, indicating the involvement of MOMCs in repair and regeneration of the damaged tissue [70,71]. Currently, several studies have cautioned us about the potential of bone marrow-derived HSCs/HPCs for muscle regeneration [48,72–74]. In addition, bone marrow-derived cells isolated from Tie2-GFP mice do not engraft into skeletal muscle microvasculature but promote angiogenesis after acute injury [75]. Furthermore, recent experiments using transgenic mice for developmental and conditional Pax7 gene knockout strongly indicates that satellite cells are a major cell source for the postnatal muscle regeneration [76,77].

## Tissue Resident HSCs

Many adult tissues besides skeletal muscle, such as brain, heart, lung, kidney, and small intestine contain different amounts of HSCs and/or HPCs that can be also enriched in the CD45(+) and SP fraction [30,34]. Therefore, the HSCs and/or HPCs are normal residents in many adult tissues and might contribute to tissue regeneration. It should be elucidated whether other adult tissue-derived HSCs/HPCs also exhibit the potential of hematopoietic reconstitution of irradiated mice. *In vitro* hematopoietic colony forming assays demonstrate that bone marrow, skeletal muscle, spleen and liver appear to contain more undifferentiated multipotential myeloid progenitors than the other tissues (brain, heart, lung, kidney, and small intestine), which contain more committed myeloid progenitors, such as macrophages and granulocytes [1,30,34]. This observation implies that there are unique characteristics about the skeletal muscle niche that allows it to support the survival and maintenance of HSCs/HPCs. Comparison of mRNA levels in skeletal muscle- and fat tissue-derived CD45(+) SP cells revealed that although they expressed many of the same genes including hematopoietic markers (CD45, CD34, CD14, CD68, Thy-1, VCAM-1 and Sca-1) and other genes (Notch, Cdkn1a/p21, Hes1 and Akt1), fat tissue-derived CD45(+) SP cells expressed higher levels of *c-kit*, whereas muscle-derived CD45(+) SP cells possessed a clear enrichment for several endothelial specific transcripts such as (Endoglin, VE-cadhein, Caveolin-1, ABCG2, PECAM and Flk-1) and other genes (Jag1 and Sparc/Osteonectin), indicating that muscle-derived CD45(+) SP cells are distinct from those isolated from fat tissue. Thus, CD45(+) SP cells do not simply represent a common pool of circulating progenitors, but seems to possess characteristics likely specified by the tissue niche in which they reside [78]. Recent work demonstrates that stem cells are closely associated with vascular niche in the tissues. For example, satellite cells are positioned in a juxtavascular manner while reciprocally interacting with endothelial cells during differentiation to support angio-myogenesis [79,80]. In addition, HSCs reside in a perivascular niche (endothelial cells and perivascular stromal cells) in which multiple cell types express factors that promote HSC maintenance [81].

## DMD Therapy by Bone Marrow Transplantation

In considering translational research of angiogenic and myogenic progenitors, biological relevance in treating muscle dystrophies is a sought after bridge to clinical application. Duchenne Muscular Dystrophy (DMD) is the most common muscular dystrophy in which mutations are found in the dystrophin gene that encodes the primary membrane anchor protein essential for skeletal muscle stability [82]. Definitive skeletal muscle treatment for muscular dystrophy will then likely require restoration of the dystrophin protein complex in all affected muscle groups. One promising approach used to restore dystrophin and regenerate muscle fibers is cell therapy. Looking at the summary of the current state of understanding for cell therapies, bone marrow or HSCs/HPCs transplantation is the potential therapeutic approach for treating muscular dystrophy. With the capacity of this therapy many groups have examined transplantation of bone marrow-derived cells into muscular dystrophy animal models. As a result, several papers showed some contributions of bone marrow-derived cell transplantation to skeletal muscle fibers in several muscular dystrophy model and spinal muscular atrophy model mice [21,83–92], while other papers reported negative results [72,93,94]. Interestingly, the Gussoni et al. [95] reported the analysis of muscle biopsies from a DMD patient who received bone marrow transplantation at age 1 year for severe combined immune deficiency. Analysis of muscle biopsies from this patient at age 12 years revealed the presence of donor nuclei within a small number of muscle myofibers (0.5–0.9%), indicating the contribution of donor-derived bone marrow cells to DMD host muscle fibers [95]. In addition, bone marrow transplantation in a human patient with Diamond-Blackfan anemia and co-existing DMD demonstrated that in a patient with 100% donor chimerism of the hematopoietic system, muscle tissue presented 8% to 10.4% of cells being of donor origin, indicating promising effects for bone marrow transplantation to muscular dystrophy [96].

The muscle-derived HSCs/HPCs are capable of differentiation into hematopoietic cells *in vitro* and *in vivo*, and thus these muscle-derived HSCs/HPCs appear to have characteristics similar to classical hematopoietic stem/progenitor cells found in bone marrow. Clearly, further experimentation is required to investigate the origin, biological significance and the cellular niche for the HSCs/HPCs within non-hematopoietic tissues. A new and encouraging route in this investigation is the possibility to find wider application for the use of muscle-derived and/or bone marrow-derivedHSCs/HPCs as a potential cell therapy for muscular dystrophy.
